# Temporal and spatial dynamics of *Cryptosporidium parvum *infection on dairy farms in the New York City Watershed: a cluster analysis based on crude and Bayesian risk estimates

**DOI:** 10.1186/1476-072X-9-31

**Published:** 2010-06-17

**Authors:** Barbara Szonyi, Susan E Wade, Hussni O Mohammed

**Affiliations:** 1 Department of Population Medicine and Diagnostic Sciences, College of Veterinary Medicine, Cornell University, Ithaca, NY, 14853, USA

## Abstract

**Background:**

*Cryptosporidium parvum *is one of the most important biological contaminants in drinking water that produces life threatening infection in people with compromised immune systems. Dairy calves are thought to be the primary source of *C. parvum *contamination in watersheds. Understanding the spatial and temporal variation in the risk of *C. parvum *infection in dairy cattle is essential for designing cost-effective watershed management strategies to protect drinking water sources. Crude and Bayesian seasonal risk estimates for *Cryptosporidium *in dairy calves were used to investigate the spatio-temporal dynamics of *C. parvum *infection on dairy farms in the New York City watershed.

**Results:**

Both global (Global Moran's I) and specific (SaTScan) cluster analysis methods revealed a significant (p < 0.05) elliptical spatial cluster in the winter with a relative risk of 5.8, but not in other seasons. There was a two-fold increase in the risk of *C. parvum *infection in all herds in the summer (p = 0.002), compared to the rest of the year. Bayesian estimates did not show significant spatial autocorrelation in any season.

**Conclusions:**

Although we were not able to identify seasonal clusters using Bayesian approach, crude estimates highlighted both temporal and spatial clusters of *C. parvum *infection in dairy herds in a major watershed. We recommend that further studies focus on the factors that may lead to the presence of *C. parvum *clusters within the watershed, so that monitoring and prevention practices such as stream monitoring, riparian buffers, fencing and manure management can be prioritized and improved, to protect drinking water supplies and public health.

## Background

*Cryptosporidium *is a protozoan parasite that is recognized as one of the most important biological contaminants in drinking water [1]. Cryptosporidiosis is associated with gastrointestinal infection which can be life threatening in immuno-compromised individuals. The infection is transmitted by the fecal-oral route either by direct contact or through contamination of food and water [2]. An experimental study of healthy adult volunteers revealed that the ingestion of as few as 30 *Cryptosporidium *oocysts can initiate infection [1]. Water-borne transmission is facilitated by the long-lasting infectivity of the oocyst in the environment and its resistance to conventional water treatment technologies such as chlorination [3].

The New York City Watershed is currently the focus of a long-term project investigating the public health risk of waterborne cryptosporidiosis. Active surveillance in the city which began in 1994 has identified over 100 cases of cryptosporidiosis annually among NYC residents [4]. A quantitative risk assessment model for cryptosporidiosis in NYC predicted that the mean annual risk estimates for infection for all ages and persons with or without HIV/AIDS exceed the proposed acceptable annual risk level of 1 case of infection per 10,000 [5].

The New York City water supply system provides drinking water to almost half the population of New York State, which includes over 8 million people in the City and one million in Upstate counties, plus millions of commuters and tourists. The water is supplied from a network of 19 reservoirs and three controlled lakes that contain a total storage capacity of approximately 2 billion cubic meters. The total watershed area for the system is approximately 5,100 km^2 ^extending over 200 km north and west of NYC. The system is dependent on precipitation and subsequent runoff via streams and rivers to supply the reservoirs. The water is then moved via a series of gravity-fed aqueducts to the distributions system, where it is chlorinated before it reaches the consumers [6]. Pathogens such as *Cryptosporidium *pose a significant threat to public health in the City's unfiltered water supply, because the oocysts are very resistant to chlorination, and they are regularly detected in reservoir effluents [3,6].

Dairy calves are thought to be a primary source of zoonotic *Cryptosporidium parvum *contamination in watershed ecosystems [7]. In the NYC Watershed, the Catskill/Delaware drainage system is home to approximately 200 dairy farms. To avoid building a huge filtration plant that could cost about $8 billion and the associated $300 million per year for operating costs, NYC implements extensive watershed management measures, including water quality monitoring and best management practices (BMP) on agricultural land, with the goal to protect water quality while maintaining economic viability on these farms [8]. Watershed management requires a network design that demands distinct spatial and temporal monitoring and protection efforts [6]. However to date, the spatial and temporal variation in the risk of *C. parvum *infection in dairy herds in watersheds has not been investigated. Understanding the spatial and temporal pattern of *C. parvum *infection on dairy farms would be useful in designing or modifying watershed management strategies to monitor and mitigate the risk of *C. parvum *contamination in watersheds.

Bayesian approach has been used increasingly in geographical epidemiologic studies, because it stabilizes crude risk estimates by reducing variance heterogeneity. Thus, risk maps based on Bayesian rather than crude risk estimates are preferred because they are more accurate and visually appealing [9]. In a Bayesian approach, a prior probability distribution for the values of a parameter (based on previous studies) is converted (under the influence of current observations) to a posterior distribution of that parameter. This posterior distribution is used to provide an estimate for the parameter [10].

The objectives of the study were to 1) explore and map the temporal and spatial dynamics of the risk of *C. parvum *infection in dairy cattle in the NYC Watershed, and to 2) identify high-risk clusters in space and time. The study utilized both crude-, and Bayesian prevalence estimates to accurately describe the spatial epidemiology of this important zoonotic parasite among dairy herds in a large watershed ecosystem.

## Methods

### Description of data and study area

The crude *C. parvum *prevalence estimates were based on a series of cross-sectional studies conducted in the Delaware portion of the NYC Watershed [11]. The study farms were located within the Cannonsville drainage basin in the City's Delaware Water Supply System, which is the largest basin in the City's system, encompassing an area of 1200 km^2 ^within Delaware County [4]. Most of the dairy farms in the study area were family operated, small-scale farms occupying an average of 1-2 km^2 ^and maintaining a herd of approximately 150 mature dairy cows. There was a year-round calving pattern and most farmers spread calf manure on their fields regularly. The majority of the farmers maintained an open herd (i.e. regularly purchased cattle from other herds). Mature cattle were kept on pasture during the summer months and often had direct access to springs. Several farms used untreated spring water as water source for the barn. The study population was drawn from dairy herds enrolled in the Watershed Agricultural Program, which is a voluntary partnership between watershed farmers and the City, aimed at developing and implementing pollution prevention plans on farms to protect water quality. Thirty-two dairy farms were visited once in each of three different seasons defined as spring (April-June) summer (July-September) and winter (December-March). A total of 507 fecal samples were collected from pre-weaned calves (with or without apparent signs of illness) and screened for the presence of *Cryptosporidium *with a quantitative centrifugation flotation method and bright-field microscopy. We considered a sample positive by flotation when at least one oocyst with the correct morphological characters was identified (*C. parvum*-like oocysts are 4-6 *μ*m and spherical; contain a residuum and sporozoites; refract pink in sugar and have a halo in phase) [12]. The protocol for the undertaken studies was approved by the Institutional Animal Care and Use Committee at Cornell (Protocol # 00-4). The number of calves examined in each season and corresponding flotation results, and prevalence estimates are summarized in Table 1.

**Table 1:** Characteristics of the data used in the study

Season	N samples^a^	N positive^b^	CP^c^	Range of CP^d^	BP^e^	Range of BP^f^
**Spring**	150	23	15	0-100	10	0-37
**Summer**	182	47	26	0-100	19	0-48
**Winter**	175	20	11	0-64	9	0-51

^a^Number of pre-weaned calves tested with the flotation method

^b^Number of pre-weaned calves positive for *C. parvum *with the flotation method

^c^Crude average prevalence of *C. parvum *among pre-weaned calves expressed as percent

^d^Range of crude within-herd prevalence of *C. parvum *expressed as percent

^e^BP Bayesian average prevalence of *C. parvum *among pre-weaned calves expressed as percent

^f^Range of Bayesian within-herd prevalence of *C. parvum *expressed as percent

### Bayesian model

Prevalence data from the cross-sectional studies described above were used to fit a hierarchical Bayesian model using WinBugs version 1.4 software [13]. All prior estimates in the model were based on the results of epidemiologic studies that our group had conducted among dairy herds in New York State watersheds [14,15]. Thus the sensitivity and specificity of the flotation method was 0.75 and 0.96, respectively [15]. The Bayesian model was based on binomial sampling (Equation 1) [16].(1)

where *y*_*t *_was the number of flotation-positive calves on farm *t*, *n*_*t *_was the total number of calves tested on farm *t*, *π*_*t *_was the prevalence of *Cryptosporidium *among pre-weaned calves on farm *t*, and Se and Sp were the sensitivity and specificity, respectively, of the flotation method. The term (*π_t_*Se + (1-*π_t_*)(1-Sp)) is the probability of a test positive result for a particular calf. Because of our concern regarding the potential over-dispersion in the estimate of *π*_t _(due to the fact that animals are clustered by farm) we controlled for this dependency by conditioning the estimate on farm to achieve approximate conditional independence. In other words, we used a hierarchical modelling approach to be able to pool the information on the prevalence of *Cryptosporidium *from these herds without assuming that they belonged precisely to the same population. To achieve approximate conditional independence, we assigned Bayesian hyperpriors to *π*_t _representing the mean *C. parvum *prevalence in pre-weaned calves in the population (*μ*), and the variability of this prevalence (*ψ*) due to aggregation by farms (Equation 2). Prior studies revealed that the average prevalence of *C. parvum *in a New York State watershed in pre-weaned cattle was 20% [14], therefore *μ *was modelled as beta (12.82, 48.28) with a most likely value of 20%. The same study revealed that the within-farm prevalence of *C. parvum *in this population ranged from 0-40%. Using this estimate, the variability among *C. parvum *prevalence in calves on different farms (*ψ*) was modelled as gamma (4.5, 0.5). Previous studies also revealed that not all herds in the study area were infected with *C. parvum *[17]. To allow for the possibility of a *C. parvum*-free herd, *π*_t _was modelled with a mixture distribution. A prior study in an adjacent watershed estimated that 42% of the herds were infected with *C. parvum *[18]. In the current study, the probability of a herd being infected (*τ*) was modelled as beta (9.51, 12.75) with a most likely value of 0.42. Thus, *π*_t _was modelled as a mixture beta-distribution with the hyperpriors *μ *and *ψ *as described in equation 2.(2)

The Bayesian prevalence estimates are summarized in Table 1.

### Potential clustering of zoonotic strains

The overall clustering tendency of the disease risk in the study region was assessed by a test of global spatial autocorrelation, which only investigates the presence but not the exact location of the cluster(s). Spatial autocorrelation arises when risk estimates from neighbouring farms are not independent, i.e., correlated. This correlation is measured using the Moran's I. High values for the I implies that disease rates for geographically closer farms are more highly correlated than those from farms that are geographically distant [19]. The Moran's I statistic is defined as follows:

where *N *is the number of farms,  is the average prevalence on the farms, *X*_*i *_and *X*_*j *_are the prevalence on farm *i *and *j*, respectively, and *W*_*ij *_is the spatial weight between farms *i *and *j*, determined by the distance between farms *i *and *j*. The Z Score associated with the index is based on the Randomization Null Hypothesis stating that "there is no spatial clustering". Thus Z-scores greater than 1.96 or smaller than -1.96 indicate significant spatial autocorrelation at the 5% level [19].

The spatial relationship among the farms was conceptualized with the inverse distance model (the impact of one feature on another decreases with distance). The global spatial autocorrelation test was performed on both the crud and the Bayesian prevalence estimates by season, using the geographical information system (GIS) software ArcView 9.2 (ESRI, CA, USA).

The scan statistic implemented in the software SaTScan v8.0.1 [20] was used to test for the presence of purely spatial, purely temporal, and space-time clusters, and to identify their location. The SaTScan statistic evaluates clusters in temporal, spatial and space-time setting by gradually scanning a window across time and/or space. Purely spatial analysis utilizes circular or oval scanning windows, while space-time analysis uses cylinders, with the base representing space and the height indicating time. For each window a likelihood ratio statistic is computed based on the number of observed and expected cases within and outside the window. The likelihood function assuming Poisson distributed cases is proportional to:

Where *N *is the total number of cases, *c *and *E*[*c*] represent the observed and expected number of cases in a window, while *N *- *c *and *N *- *E*[*c*] indicate the observed and expected number of cases outside the window. The indicator function *I*() is equal to 1 when the window has more cases than expected under the null hypothesis, and zero otherwise. The window with the highest likelihood ratio is the most likely cluster and is assigned a p value through 999 Monte Carlo simulations [21]. A Poisson model was fitted to the raw data for each season to examine the presence of purely spatial clusters. A Poisson model was also applied to the entire dataset to determine whether purely temporal (i.e. seasonal) or space-time clusters existed during the course of a year. At each farm location, cases were defined as the number of calves that tested positive for *Cryptosporidium *by the flotation method, while the population size was the total number of calves that were tested. The maximum cluster size was set at the recommended value (50% of the total population at risk). Both circular and oval cluster shapes were evaluated.

### Mapping the risk of *C. parvum *in the watershed

Geospatial coordinates for each farm were collected with a Garmin eTrex Summit handheld global positioning system (GPS) device (Garmin International Inc, Olathe, Kansas, USA) and imported into the GIS software Manifold System 8.0 Ultimate Edition (Manifold, Carson City, NV, USA). The geographical coordinates were re-projected into the Universal Transverse Mercator coordinate system, Zone 18(N), North American Datum 1983, and overlaid with the shapefile of Delaware County, NY obtained from the New York State Geographic Information System Clearinghouse http://www.nysgis.state.ny.us. Dot maps indicating the seasonal prevalence estimates on the study farms were created to examine the spatial dynamics of *C. parvum *in the watershed during the annual cycle, and to explore the differences between the crude and the Bayesian estimates.

## Results

### Potential clustering of zoonotic strains

The Global Moran's I statistic was performed on both crude and Bayesian prevalence estimates to determine the presence of global autocorrelation in three different seasons. The results of this analysis are summarised in Table 2. The crude estimates revealed significant spatial autocorrelation in the winter with a Global Moran's I value of 0.18 (p = 0.03), and no clustering in the spring and summer. In contrast, the Bayesian prevalence estimates did not show significant overall clustering tendency in any of the seasons examined.

**Table 2:** Results for the test of global spatial autocorrelation using Moran's I statistics based on crude-, and Bayesian risk estimates

Season	Estimate	Moran's I	Z-score	p-value
**Spring**				
	Crude	0.0087	0.41	0.67
	Bayesian	-0.0067	0.24	0.8
**Summer**				
	Crude	-0.17	-1.4	0.15
	Bayesian	-0.1	-0.64	0.51
**Winter**				
	Crude	0.18	2.07	0.03
	Bayesian	0.061	1.03	0.3

**Figure 1:**
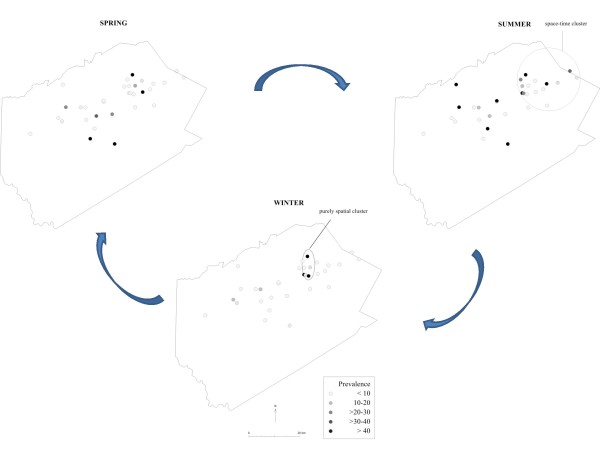
**Dot maps of the spatio-temporal dynamics of *C. parvum *infection in dairy herds in Delaware County, NY, based on A, raw prevalence estimates. Significant circular clusters identified by the SaTScan statistics are also shown**.

Crude prevalence estimates were used to test for the presence of spatial, temporal, and space-time clusters using the Poisson model with the scan statistic. The results of the SaTScan analyses are summarized in Table 3 and the significant (p < 0.05) spatial and space-time clusters are shown in Figure 1. The purely temporal analysis revealed a 2-fold increase in the risk of *C. parvum *infection in the summer affecting all the herds in the study area. A significant (p = 0.003) oval-shaped cluster was identified in the winter, with a more than 5-fold increase in risk inside the cluster compared to the rest of the study area. In addition, significant space-time clusters were detected in the summer with both oval and circular scanning window settings. These space-time clusters included nearly 50% of the population at risk and overlapped geographically. Therefore, only the circular space-time cluster is shown in Figure 1.

**Table 3:** The most likely temporal, spatial, and space-time clusters identified by the SaTScan statistics using the Poisson probability model, based on crude prevalence estimates

Analysis type	Observed^a^	Expected^b^	RR^c^	P-value	Population^d^	Shape
**Purely Temporal**	57	38	2	0.002	169	
**Purely Spatial**						
Spring	no significant cluster					
Summer	no significant cluster					
Winter	14	4	5.87	0.003	29	elliptical
**Space-Time**	37	20	2.3	0.031	83	circular
	40	17	3	0.004	78	elliptical

^a^Number of observed cases in cluster

^b^Number of expected cases in cluster

^c^Relative Risk

^d^Number of animals at risk in the cluster

### Risk maps

**Figure 2:**
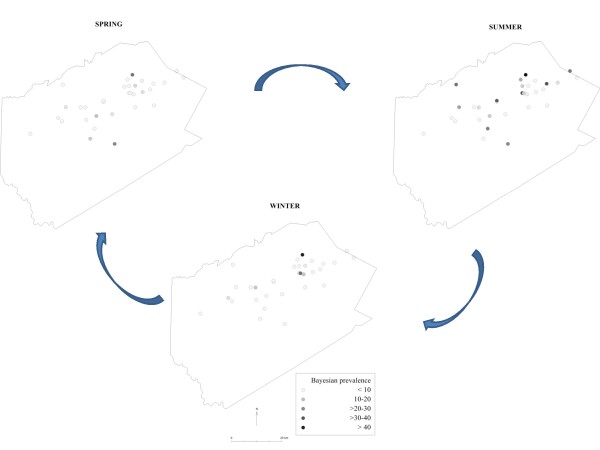
**Dot maps of the spatio-temporal dynamics of *C. parvum *infection in dairy herds in Delaware County, NY, based on Bayesian estimates**.

Figures 1 and 2 are dot maps of the spatio-temporal dynamics of *C. parvum *infection in dairy herds in Delaware County, NY, based on crude and Bayesian prevalence estimates, respectively. The significant spatial and space-time clusters identified by the SatScan statistics are also indicated. Although cluster analyses based on Bayesian estimates did not show significant spatial clustering in any season, the map revealed a diffuse increase in the risk of *C. parvum *contamination in the summer.

## Discussion

We carried out this study to evaluate potential clustering of dairy herds that are infected with *C. parvum *in the NYC Watershed. This was the first study to evaluate the spatial and temporal variation in the risk of *C. parvum *infection in dairy cattle in an important watershed.

The decision to include only pre-weaned calves in this study was based on the results of a quantitative risk assessment (QRA) of *Cryptosporidium *in dairy cattle in the NYC Watershed which revealed, that despite representing only a small proportion of the population and producing a small fraction of manure, pre-weaned calves produced the vast majority of all zoonotic *C. parvum *oocysts shed within the dairy cattle population. Specifically, it was estimated that pre-weaned calves produced 99.5% of the total *C. parvum *oocyst burden with a calculated mean log oocyst shedding of 4.02 × 10^10 ^daily. Thus it was estimated that pre-weaned calves produce nearly all the *C. parvum *oocysts that contaminate the watershed [15].

One of the assumptions we made in the models was that all *C. parvum*-like oocysts shed by pre-weaned calves were zoonotic. We made this assumption because molecular typing is required to determine the zoonotic potential of *C. parvum*-like oocysts, and this necessity would have further amplified the problem of small numbers. While over-estimating the zoonotic risk, we felt this assumption was reasonable, because recent studies that applied molecular typing revealed that the majority of *Cryptosporidium *infections in pre-weaned calves were indeed zoonotic [11,18,22].

Both global (Moran's I) and specific (SaTScan) cluster detection methods identified a significant spatial cluster of *C. parvum *infection in calves in the winter, with a relative risk (RR) of 5.8, based on crude risk estimates. No other purely spatial clusters were identified with either method. Thus, there was complete agreement between the results of the two cluster detection methods. In addition, the scan statistics detected a significant space-time cluster in the summer with both circular (RR = 2.3) and elliptical (RR = 3) window settings. Further investigation revealed the presence of a significant temporal cluster (RR = 2) but the lack of a purely spatial cluster in the summer, which suggests that the space-time clusters identified in the summer were due to a temporal rather than a spatial increase in risk. The large sizes of the space-time clusters including nearly 50% of the population at risk (maximum allowed under the conditions specified) also supports the notion of a spatially diffuse increase in the risk of *C. parvum *infection in the summer.

It has been suggested that farms downstream of other farms may be contaminated with *Cryptosporidium *via runoff from farms upstream, although evidence for this epidemiologic link is lacking [23-25]. The rationale for considering elliptical spatial clusters in this study was that farm-to-farm transmission via runoff would be expected to produce an elliptical rather than a circular cluster.

The term disease cluster is defined as an increase in the expected number of cases within a population bounded in space and time [26]. We used two different cluster detection methods to ensure comparability and robustness of results. We elected to use the scan statistics for the investigation of temporal and space-time clusters, because recent studies identified SatScan as the most developed and robust space-time surveillance software package that takes multiple testing problems into account, and is considered the most powerful for detecting localized clusters [27].

The major limitation of the study was the low number of pre-weaned calves on the farms, resulting in unstable risk estimates as small populations have large variability in rates [28]. For example, the small number of cases and population at risk may have accounted for the high relative risk estimate (RR = 5.8) for the winter spatial cluster. This limitation was corrected with the use of a Bayesian approach that has the ability to stabilize the raw estimates derived from a small number of individuals [29]. The Bayesian approach also allowed us to incorporate prior knowledge about the risk of *C. parvum *infection in the target population, and to account for the imperfections of the diagnostic test. However, the quality of this prior information might influence the quality of the estimate and hence could be a source of bias and limitations [26]. The degree of smoothing provided by the Bayesian approach is a trade-off between high sensitivity (truly high risk areas correctly identified) and high specificity (areas without excessive risk correctly identified) such that sensitive but non-specific measure will generate many false positive findings, whereas a specific but not sensitive measure will miss areas with high risk [26]. In our study we encountered contradictory results in the analysis of purely spatial clusters using Global Moran's I statistics. While crude estimates revealed significant spatial autocorrelation in the winter, Bayesian estimates indicated the lack of a spatial clustering in any season. Considering the limitations of using crude vs. Bayesian estimates in spatial analysis, the discrepancy in our results may be due either to the instability of the crude estimates leading to spuriously high values, or to the low sensitivity of the Bayesian model to detect areas with truly high risk.

*Cryptosporidium *is considered a non-point source pollutant in watersheds that is carried off the land surface during rain events [30,31]. Monitoring of stream sites in the study area revealed that event based (e.g. after storms) *Cryptosporidium *concentrations were consistently higher than baseline results (up to 11.7 oocysts 50L^-1^), implicating runoff as contamination source [4]. The close relationship between activities in the drainage basin and the quality of its water resources forms the underlying premise for all watershed management programs [8]. Best management practices that protect water supply on farms such as fencing, filter strips, stream crossings, animal trails and walkway, manure composting facility, and runoff management systems would ideally and ultimately be implemented on every farm in the watershed. However, until that goal is achieved, prioritization methodologies to address non-point source pollutants need to be developed, and the identification of "hot spots" is an integral part of this process.

The occurrence of spatial or temporal clusters may be due to rapid spread between locations in the case of a highly contagious disease, or the presence of common environmental risk factors [32]. The higher risk of *C. parvum *infection on dairy farms in the summer throughout the study area may be due to climatic or management factors that affect the entire area. This finding suggests that spreading manure in the summer (compared to other seasons) in any area of the watershed is associated with an increased risk of *C. parvum *contamination of the water supply. This finding is important because farms in the study area regularly spread untreated calf manure in the fields. The current recommendation is to avoid spreading manure in the spring and during frozen conditions, while summer is considered a lower risk period [8]. If further studies confirm an increased risk of *Cryptosporidium *contamination in the summer, this knowledge will be useful to improve Nutrient Management Plans, which give recommendations about the most environmentally safe time and place to spread manure.

With the City's population expected to rise to 9.1 million by 2039 from 8.3 million in 2005, watershed management will continue to have an important part to play in protecting water quality [8]. Over time, systematic and careful monitoring of disease-causing organisms and pollutants will determine the effectiveness of New York City's protection strategies and the continued success of its filtration avoidance plan. The identification of spatial or temporal "hot-spots" of *C. parvum *contamination within the watershed will have important implications for watershed monitoring and management, and need to be the focus of future investigations.

## Conclusions

The identification of *C. parvum *clusters is a priority in designing cost-effective and targeted watershed management practices to ensure safety of the water supplies for public health. This study identified high risk clusters of *C. parvum *infection in dairy herds in both space and time in a large and important watershed, suggesting that further studies are needed to determine whether the presence of clusters are persistent and predictable. We recommend that future studies focus on the causes of these "hot spots" so that watershed monitoring and management strategies may be implemented and targeted to effectively decrease *C. parvum *contamination of the water supply.

## Competing interests

The authors declare that they have no competing interests.

## Authors' contributions

BS carried out the sample collection, molecular analyses, geographical analyses including cluster detection, and drafted the manuscript. HOM conceived of, designed and coordinated the study, and helped to draft the manuscript. SEW carried out the flotation and microscopic examination of specimens and provided writing assistance. All authors read and approved the final manuscript.
